# The Institutional Worker

**Published:** 1912-07-27

**Authors:** 


### THE INSTITUTIONAL WORKER

**Being a Special Supplement to "The Hospital"**

### Editor's Notices.

**Contributions:**

Contributions should be written, or preferably typed, on one side of the paper only, and all articles sent in are accepted upon the distinct understanding that they are forwarded to The Hospital only.

The Editor cannot undertake to return MSS. not used, but when a stamped directed envelope is enclosed the MSS. may be returned if a special request is made.

Accepted articles and paragraphs of news will be paid for after publication at the scale rate.

**Address:**

To prevent delay all contributions and letters on editorial business must be addressed exclusively to the Editor, "The Hospital," 29 Southampton Street, Strand, London, W.C. It is important that this regulation shall be strictly observed.

**Correspondence:**

Correspondence on all subjects is invited. The name and address of correspondents must be given as a guarantee of good faith, but not necessarily for publication.

**Special Articles:**

Special articles are invited, and questions, inquiries, and paragraphs upon all matters relating to the work, administration, and management of general, special, mental, fever, cottage and convalescent hospitals, sanatoria, homes, institutions, societies and organisations for the treatment or care of the sick, injured and dependants of all classes. Every matter affecting the interests and welfare of the staff of all grades working in these institutions will receive special consideration.

**Administrative Medicine, Research and Items of News:**

Special terms will be paid for approved articles on subjects in this wide field by experts who have made some section of it their special study and interest. We wish to give prominence to every movement tending to advance accuracy of diagnosis, efficiency in treatment, and the development of modern methods to eradicate disease and promote the welfare of the suffering.

**A Bureau of Information:**

We invite inquiries and applications for information and help in providing for that numerous class of sufferers who require special care or house-room which they cannot obtain in their own homes or secure for themselves. **We cannot, however, prescribe, or recommend practitioners.**

**Books for Review:**

Publishers are particularly requested to send advance proofs of any new books of importance whenever possible, as the Editor has made arrangements to publish immediate reviews on a new plan.

**Photographs, Plans, Blocks, and Illustrations:**

It is requested that wherever possible MSS. may be accompanied by illustrations in any of the above forms. The name of the sender and of the article to which it belongs should be written on the back of each photograph, drawing, or block for purposes of identification.

**Local Papers:**

Newspapers containing reports or news paragraphs **should be marked** and addressed to the Sub-Editor.

**The Coupon System:**

A coupon will be found at the bottom of the third inside page of the cover each week. This coupon must be attached to every question or inquiry to which an answer is desired in The Hospital.

### AN INVITATION

**The Bureau of Information instituted by "The Hospital" is open to every reader of the Journal and enquiries on all subjects are cordially invited.**

THE MANY DIFFICULTIES therefore that have hitherto presented themselves to Institutional Workers seeking accurate information on any phase of Institutional Life and Work ARE NO LONGER A BAR TO PROGRESS.

THE ACCUMULATION OF YEARS OF EXPERIENCE in Institutional Affairs and in every Department of the Medical, Health and Sanitary Services IS AT THE DISPOSAL OF ANY READER REQUIRING INFORMATION OR HELP.

EVERY QUESTION IS DEALT WITH BY AN EXPERT who is best able to cope with the particular problem upon which assistance is required. THE UTMOST RELIANCE CONSEQUENTLY CAN BE PLACED IN THE INFORMATION SUPPLIED.

THE NUMEROUS AND VARIED COMMUNICATIONS RECEIVED DAILY from all parts of the Kingdom fully justify the new department and ARE ELOQUENT TESTIMONY OF THE FACT THAT "THE HOSPITAL" BUREAU OF INFORMATION SUPPLIES A LONG-FELT WANT.

**All letters regarding the Bureau MUST BE ADDRESSED to "The Hospital" Bureau of Information, "The Hospital" Building, 28-29 Southampton Street, Strand, London, W.C.**

### The Scientific Press Publications

### INSTITUTIONAL WORKS.

### HOSPITALS AND ASYLUMS OF THE WORLD.

**By SIR HENRY BURDETT, K.C.B., K.C.V.O.**

In Four Volumes, with Separate Portfolio containing several hundred Plans.

**Vols. I. and II. (ASYLUMS)** ... **Price £6 15s.** | **Vols. III. and IV. (HOSPITALS) and the Portfolio of Plans** ... **Price £9**

**The Portfolio of Plans** (20 in. × 14 in., drawn to uniform scale), **Price £4 14 6**

**The Complete Work** ... ... **£12 12s.**

This magnificent work forms a Complete Survey of the Origin, History, Construction, Administration and Management of the Hospitals and Asylums of the World, with detailed information concerning the principal Institutions.

*Only a few copies remain unsold.*

### LEDGERS, ACCOUNT BOOKS, STOCK BOOKS of all kinds for large and small Institutions.

These are supplied already ruled and printed, or in accordance with the special requirements of institution managers.

### ANNUAL REPORTS AND PROSPECTUSES.

THE SCIENTIFIC PRESS are prepared to undertake the production of the Annual Reports and Prospectuses of every class of Institution, and to supply printed or typewritten copies of testimonials for the officials. Professional Cards.

**THE SCIENTIFIC PRESS, LTD.,**
**28 and 29 Southampton Street, Strand, London, W.C.**

SEND FOR COMPLETE CATALOGUE.

NOW READY. Price 10/6 net; 10/11 Post Free.

### Burdett's Hospitals & Charities, 1912.

### THE YEAR BOOK OF PHILANTHROPY, AND HOSPITAL ANNUAL.

**Edited by SIR HENRY BURDETT, K.C.B., K.C.V.O.**

*The volume for* 1912 *contains Special Chapters, with carefully analysed figures, on :*

The Volume of Charity.
Voluntary Hospitals in 1911.
The King's Fund and the League of Mercy.
The National Insurance Act and the Voluntary Hospitals.
Hospital Construction, 1911.
The Nursing Department and its Cost.
Hospital Sunday.
Hospital Saturday.
Home and Foreign Missions.
Convalescent Homes.
Orphanages, Homes and Charities.
Hospital Finance—Income in 1910.
Hospital Expenditure—Expenditure in 1910.
Income and Expenditure in Typical Hospitals.
Canada, Australia and India.

In addition to a Complete Directory of Hospitals and Charitable Institutions throughout the English-speaking World

"The ideal of what a work of reference ought to be."—*The Times.*

Of all Booksellers, or of

**THE SCIENTIFIC PRESS, LTD., 28 & 29 Southampton Street, Strand, LONDON, W.C.**

